# Prevalence and associated factors of insomnia symptoms among doctors and nurses under the context of high prevalence of multiple infectious diseases: a cross-sectional study

**DOI:** 10.3389/fpubh.2024.1423216

**Published:** 2024-08-29

**Authors:** Xu Chen, Bin Wang, Yu Wang, Surui He, Dongmei Liu, Dongmei Yan

**Affiliations:** ^1^Lianyungang Maternal and Child Health Hospital, Lianyungang, China; ^2^Department of Nursing, The First Affiliated Hospital of Dalian Medical University, Dalian, China; ^3^Department of Nursing, The Second Affiliated Hospital of Dalian Medical University, Dalian, China

**Keywords:** insomnia symptoms, associated factors, doctors, nurses, China

## Abstract

**Introduction:**

Insomnia symptoms are prevalent among healthcare workers and represent a potential public health problem. However, there is currently insufficient evidence on insomnia symptoms among doctors and nurses under the context of high prevalence of multiple infectious diseases after the pandemic in China. The purpose of this study was to analyze the prevalence of insomnia symptoms among doctors and nurses in third-grade class-A general hospitals under the context of high prevalence of multiple infectious diseases, and to explore the influence of demographic characteristics, work-related factors, health and lifestyle-related factors on insomnia symptoms.

**Methods:**

An institution-based cross-sectional survey was conducted among doctors and nurses in two third-grade class-A general hospitals. A structured questionnaire was used to collect information on demographic characteristics, work-related factors, health and lifestyle-related factors, and insomnia symptoms among doctors and nurses. Multivariate logistics regression analysis was applied to identify factors significantly associated with insomnia symptoms among doctors and nurses, respectively.

**Results:**

A total of 1,004 participants were included in this study, including 503 doctors and 501 nurses. The prevalence of insomnia symptoms in doctors and nurses was 47.7 and 51.3%, respectively. Multivariate logistics regression analysis showed that workplace violence (*OR*: 1.631, 95% *CI*: 1.050–2.532), doctor-patient relationship (*OR*: 1.603, 95% *CI*: 1.049–2.450), chronic pain (*OR*: 4.134, 95% *CI*: 2.579–6.625), chronic disease (*OR*: 1.825, 95% *CI*: 1.164–2.861), and anxiety symptoms (*OR*: 2.273, 95% *CI*: 1.357–3.807) were associated factors with insomnia symptoms in doctors. Education (*OR*: 0.301, 95% *CI*: 0.106–0.851), service years (*OR*: 1.978, 95% *CI*: 1.304–3.002), weekly working hours (*OR*: 1.694, 95% *CI*: 1.061–2.705), chronic pain (*OR*: 5.359, 95% *CI*: 3.241–8.860), and anxiety symptoms (*OR*: 2.472, 95% *CI*: 1.478–4.136) were associated factors with insomnia symptoms in nurses.

**Conclusion:**

The prevalence of insomnia symptoms among doctors and nurses was high, and affected by many factors. This information can inform tailored interventions to insomnia symptoms by doctors and nurses who play an important role in public health.

## Introduction

Sleep is an irreplaceable form of rest that allows the body to function undisturbed and is an important determinant of the level of health (1). Sleep problems have negative effects on the immune system and metabolism and are also associated with diseases such as hypertension (2, 3). In addition, insomnia symptoms affect concentration and are a risk factor for excessive daytime sleepiness (4, 5). Sleep problems also have significant economic costs. The Australian study showed that the financial cost of sleep disorders was equivalent to 0.73% of gross domestic product, and the non-financial cost was 3.2% of the total burden of disease for the year (6).

Healthcare workers were at higher risk for sleep quality problems such as insomnia symptoms than other occupational groups (7, 8). The level of insomnia symptoms in nurses was significantly higher than the normal level in the general population (9). A qualitative analysis of nurses revealed that 92% of participants reported at least one sleep problem, of which 68% were insomnia-related problems (10). A systematic review and meta-analysis revealed an estimated prevalence of insomnia symptoms of healthcare workers during the COVID-19 pandemic of 38.9% (11). Given the prevalence of insomnia symptoms among healthcare workers, it is necessary to develop possible mitigation strategies. One study showed that 95% of nurses expressed interest in participating in sleep interventions (10). Improving insomnia symptoms may provide important benefits to the health of healthcare workers and healthcare system. However, the development of strategies to improve insomnia symptoms first requires the identification of risk factors for insomnia symptoms.

Previous studies among doctors and nurses identified a number of risk factors for insomnia symptoms. Studies in China found that organic diseases were independent factors for insomnia symptoms in healthcare workers (8). Studies in Bangladesh revealed a higher risk of severe insomnia symptoms among female healthcare workers (12). Studies in Greek showed that young nurses were prone to insomnia (13). Studies in Brazilian nurses showed that job strain was associated with increased odds of insomnia symptoms. The prevalence of insomnia symptoms was higher among nurses with particularly low or high body mass index (BMI), no physical activity, high-risk alcohol use, current smoking, and more than 3 cups of coffee per day (14). Studies in Australian nurses found that anxiety and depressive symptoms were significantly associated with insomnia symptoms (9). Studies among both doctors and nurses showed that exposure to workplace violence had a significant influence on sleep quality (15, 16). Although several studies have been published on factors associated with insomnia symptoms among doctors and nurses, there is a lack of studies that simultaneously focus on demographic characteristics, work-related factors, health and lifestyle-related factors, etc. In addition, studies in other populations had shown that pain was positively associated with insomnia symptoms and that family function could help with insomnia symptoms remission (17, 18). However, there is limited evidence regarding the influence of pain and family function on insomnia symptoms among doctors and nurses. We inferred that they might also have an influence on insomnia symptoms among doctors and nurses.

In general, previous evidence on insomnia symptoms has focused more on nurses or healthcare workers as a whole. There is a lack of research on the prevalence and influencing factors of insomnia symptoms among doctors. However, doctors and nurses differ in their duties, healthcare tasks, and hours of work, among others. It is necessary to pay attention to the insomnia symptoms and associated factors of doctors and nurses in the same context. In addition, most studies were conducted during the COVID-19 pandemic, and there was insufficient evidence on insomnia symptoms among doctors and nurses under the context of high prevalence of multiple infectious diseases after the pandemic. Previous study showed no significant change in insomnia symptoms among doctors and nurses 1 year after the end of quarantine (19). At the same time, there is a lack of data on this topic in northeast China. However, there is a large geographic variation in the prevalence of insomnia symptoms (20). It is worth noting that third-grade class-A general hospitals have higher requirements on the professional level, technical ability, academic research, innovation ability and service quality of doctors and nurses than other types of hospitals. However, there is also a lack of information on insomnia symptoms among doctors and nurses in third-grade class-A general hospitals.

In view of this, this study conducted a cross-sectional survey among doctors and nurses in two large third-grade class-A general hospitals in Dalian, Liaoning Province, Northeast China. It aimed to analyze the prevalence of insomnia symptoms among doctors and nurses in third-grade class-A general hospitals under the context of high prevalence of multiple infectious diseases, and to explore the influence of demographic characteristics, work-related factors, health and lifestyle-related factors on insomnia symptoms. This study aimed to assess insomnia symptoms among doctors and nurses separately as important evidence to guide the improvement of insomnia symptoms among doctors and nurses.

## Methods

### Study design

An institution-based cross-sectional survey was conducted from February to March 2024 in two large third-grade class-A general hospitals in Dalian, Liaoning Province, Northeast China. The two hospitals have strong technical strength and modern medical equipment, and are the university affiliated hospitals with strong comprehensive strength and reliable by the people in Liaoning region.

### Participants

Doctors and nurses who met the inclusion criteria and worked in two large third-grade class-A general hospitals were recruited using random sampling. The inclusion criteria were as follows: (1) age ≥ 18 years; (2) the length of service was greater than or equal to 1 year; (3) no history of psychiatric diagnosis; (4) consent to participate in the study. Written informed consent was obtained after a brief explanation to the participants by trained research staff. Also, a self-report questionnaire was distributed to each participant, and the participants were asked to answer each question as honestly and accurately as possible. Participation in the study was anonymous and voluntary, and all questionnaires were completed independently by the participants. The minimum required sample size was obtained using the single population proportion formula. Due to the lack of previous similar studies at this study site, in order to calculate the maximum sample size, a prevalence of insomnia symptoms as 50, 95% confidence interval (*CI*), a margin error of 5%, and a non-response rate of 20% were used in the current study. The minimum sample size required for the calculation was 461. Considering invalid questionnaires and to obtain more reliable conclusions, we decided to recruit 510 doctors and 510 nurses each. In this study, 1,020 questionnaires were distributed and all of them were recovered, with a recovery rate of 100%. Sixteen questionnaires with missing or identical answers to each question were excluded. Finally, 503 doctors and 501 nurses were included in the statistical analysis, with participation rates of 98.6 and 98.2%, respectively.

### Date collection

A structured questionnaire designed after literature review and expert consultation was used for data collection in this study. This structured questionnaire was initially pre-surveyed at the study site and was modified and adjusted. The questionnaire included demographic characteristics, work-related factors, health and lifestyle-related factors, and insomnia symptoms.

### Demographic characteristics

Gender, age, marital status, education, and professional title were collected in this study. Age was divided into three ranges including “<30,” “30–40,” and “>40.” Options for marital status included “unmarried,” “married,” and “divorced or loss of spouse.” Education was categorized as “bachelor degree or below” and “master degree or above.” Options for professional title included “no or junior,” “intermediate,” and “senior.”

### Work-related factors

Five work-related factors were assessed, including service years, weekly working hours, number of night shifts per month, workplace violence, and doctor-patient relationship. Service years were divided into “≤10” and “>10.” Weekly working hours were divided into “≤40” and “>40.” The number of night shifts per month was divided into “≤3” and “>3.”

Workplace violence was measured using the Workplace Violence Scale (WVS). It was used to assess the frequency of workplace violence experienced by healthcare workers in the past year and has been shown to have satisfactory reliability and validity (21). It consisted of 5 items: physical assault, emotional abuse, threats, verbal sexual harassment, and sexual abuse. The scores of the 5 items were summed to obtain a total WVS score, which ranged from 0 to 15. The higher the score, the higher the frequency of workplace violence experienced by the participant. A total score of 0 was defined as no violence, and a score of 1–5, 6–10, and 11–15 was defined as low, moderate, and high violence, respectively (22). In this study, a score greater than 0 was considered as having experienced workplace violence. In the study context, the Cronbach’s *α* of the WVS was 0.746.

Doctor-patient relationship was measured using the 10-item Difficult Doctor-Patient Relationship Questionnaire (DDPRQ-10). It was a widely used tool to assess the quality of the doctor-patient relationship from a doctor’s perspective (23, 24). It has already shown satisfactory reliability and validity in the Chinese healthcare setting (25). The DDPRQ-10 consisted of 10 items, each rated on a 5-point Likert scale ranging from 1 (not at all) to 5 (very much). Total scores ranged from 10 to 50, with higher scores indicated a more difficult doctor-patient relationship. In the current study, total scores greater than or equal to the mean were labeled as having a poor doctor-patient relationship (26). In the study context, the Cronbach’s α of the DDPRQ-10 was 0.815.

### Health and lifestyle-related factors

Six health and lifestyle-related factors were assessed, including chronic pain, chronic disease, physical exercise, perceived stress, anxiety symptoms, and family function. Chronic pain was measured by asking “Have you had persistent pain in a certain part of your body for at least 1 month?.” Chronic diseases were obtained by asking “Do you have a chronic disease?.” Physical exercise was categorized as “never (0 times/week),” “sometimes (1–3 times/week),” and “often (>3 times/week)” according to the number of weekly exercises (at least 30 min).

Perceived stress was measured using the Perceived Stress Scale-10 (PSS-10) (27). It measured the level of perceived stress of the study subjects in the past month. It consisted of 10 items, each with a 5-point Likert scale ranging from 0 (never) to 4 (almost always). The scores of the 10 items were summed to obtain the total PSS-10 score, which ranged from 0 to 40. Higher scores indicated higher perceived stress among participants. A total score of 15 or greater was defined as perceived higher stress (28). The PSS-10 had been widely used in China and showed good internal consistency (29, 30). In the study context, the Cronbach’s *α* of the PSS-10 was 0.768.

Anxiety symptoms were measured using the Generalized Anxiety Disorder-7 (GAD-7) (31). It assessed the frequency of anxiety symptom distress in participants over the past 2 weeks. It contained 7 items, each with a Likert scale ranging from 0 (not at all) to 3 (almost every day). The scores of the 7 items were summed to obtain the total GAD-7 score, which ranged from 0 to 21. The higher the score, the higher the degree of anxiety symptoms present by the participant. A total score of 7 or greater was considered the presence of anxiety symptoms (32). The GAD-7 had been widely used and proved to be effective (33, 34). In the study context, the Cronbach’s α of the GAD-7 was 0.928.

Family function was measured using the Family APGAR index (APGAR) (35). The scale consisted of 5 items: adaptation, partnership, growth, affection, and resolve. Five self-reported items were rated using a 3-point Likert scale, with scores ranging from 0 (hardly never) to 2 (always). Total scores varied between 0 and 10, with higher scores reflected better family function. Total scores of 0–6 and 7–10 indicate severe or moderate family dysfunction and good family function, respectively (36). The APGAR had recognized reliability and validity (37). In the study context, the Cronbach’s *α* of the APGAR was 0.915.

### Insomnia symptoms

Insomnia symptoms were measured using the Insomnia Severity Index (ISI) (38). This scale used a 5-point Likert scale to rate 7 self-reported items. The total score was the sum of the scores for each item, ranging from 0 to 28. Higher total scores represented more severe insomnia symptoms. The recommended cut-off value of the scale for detecting insomnia symptoms was 7 (39). Therefore, a total score higher than 7 was considered to have insomnia symptoms in this study. The ISI had already shown good reliability and validity (40, 41). In the study context, the Cronbach’s *α* of the ISI was 0.926.

### Data analysis

Epidata version 3.1 (EpiData Association, Odense, Denmark) software was used for data entry and SPSS version 21.0 (IBM Corporation, Armonk, State of New York) software was used for statistical analysis. Continuous variables were described as means and standard deviations, and categorical variables as frequencies and percentages. Chi-square test and Fisher’s exact test were used to compare the distribution characteristics of insomnia symptoms. Variables with *P* less than 0.05 in the Chi-square test and Fisher’s exact test were included in the multivariate logistics regression model to identify the independent associated factors with insomnia symptoms in doctors and nurses. Odds ratio (*OR*) and its 95% *CI* were calculated. Collinearity tests were performed to test for collinearity between the variables included in the regression model. It was found that the variance inflation factor of each variable was less than 10, and the tolerance was much greater than 0.1. Thus, collinearity between variables was not present. All statistical tests in this study were two-sided, and all hypotheses were tested at the 0.05 significance level.

## Results

### Characteristics of the participants

A total of 1,004 participants were included in this study, including 503 doctors and 501 nurses. In terms of demographic characteristics, the mean age of the participants was 34.96 ± 8.41 years, and more than half of the participants (50.2%) were 30 to 40 years of age. Nearly three quarters of the participants (72.7%) were female, and nearly two-thirds (64.9%) were married. The majority of participants (61.4%) had a bachelor degree or below, and almost one-fifth (20.5%) had a senior title. In terms of work-related factors, a small number of participants (39.4%) had worked for more than 10 years. More than half of the participants (56.9%) worked 40 h or less per week, and almost half (52.3%) worked more than 3 night shifts per month. More than a quarter of participants (28.2%) had experienced workplace violence in the past year, and 53.6% had a poor doctor-patient relationship. In terms of health and lifestyle-related factors, a large proportion of participants (70.2%) had pain in at least one body part for more than a month, and a minority (29.6%) had chronic disease. Only 18.9% of the participants often participated in physical exercise, and more than half (51.4%) perceived high stress. More than a quarter of participants (27.3%) had anxiety symptoms, and almost one-third (32.2%) had a moderate or severe family dysfunction (Table 1).

**Table 1:** Characteristics of the participants (*N* = 1,004).

Variables	Total (*N* = 1,004)		Doctors (*N* = 503)		Nurses (*N* = 501)	
	*N*	%	*N*	%	*N*	%
Demographic characteristics						
Gender						
Male	274	27.3	240	47.7	34	6.8
Female	730	72.7	263	52.3	467	93.2
Age (years)						
<30	283	28.2	115	22.9	168	33.5
30–40	504	50.2	231	45.9	273	54.5
>40	217	21.6	157	31.2	60	12.0
Marital status						
Unmarried	340	33.9	160	31.8	180	35.9
Married	652	64.9	339	67.4	313	62.5
Divorced or loss of spouse	12	1.2	4	0.8	8	1.6
Education						
Bachelor degree or below	616	61.4	139	27.6	477	95.2
Master degree or above	388	38.6	364	72.4	24	4.8
Professional title						
No or junior	399	39.7	158	31.4	241	48.1
Intermediate	399	39.7	154	30.6	245	48.9
Senior	206	20.5	191	38.0	15	3.0
Work-related factors						
Service years						
≤10	608	60.6	312	62.0	296	59.1
>10	396	39.4	191	38.0	205	40.9
Weekly working hours						
≤40	571	56.9	211	41.9	360	71.9
>40	433	43.1	292	58.1	141	28.1
Number of night shifts per month						
≤3	479	47.7	293	58.3	186	37.1
>3	525	52.3	210	41.7	315	62.9
Workplace violence						
No	721	71.8	343	68.2	378	75.4
Yes	283	28.2	160	31.8	123	24.6
Doctor-patient relationship						
Good	466	46.4	239	47.5	227	45.3
Poor	538	53.6	264	52.5	274	54.7
Health and lifestyle-related factors						
Chronic pain						
No	299	29.8	158	31.4	141	28.1
Yes	705	70.2	345	68.6	360	71.9
Chronic disease						
No	707	70.4	354	70.4	353	70.5
Yes	297	29.6	149	29.6	148	29.5
Physical exercise						
Never	370	36.9	159	31.6	211	42.1
Sometimes	444	44.2	224	44.5	220	43.9
Often	190	18.9	120	23.9	70	14.0
Perceived stress						
Low	488	48.6	259	51.5	229	45.7
High	516	51.4	244	48.5	272	54.3
Anxiety symptoms						
No	730	72.7	378	75.1	352	70.3
Yes	274	27.3	125	24.9	149	29.7
Family function						
Good	681	67.8	340	67.6	341	68.1
Moderate or severe dysfunction	323	32.2	163	32.4	160	31.9

### Univariate analysis of insomnia symptoms among doctors and nurses

Among 1,004 participants, the mean score of insomnia symptoms was 7.62 ± 5.68, and the prevalence of insomnia symptoms was 49.5%. The mean scores of insomnia symptoms of doctors and nurses were 7.37 ± 5.49 and 7.88 ± 5.85, respectively, and the prevalence of insomnia symptoms was 47.7 and 51.3%, respectively. Table 2 shows the associations between demographic characteristics, work-related factors, health and lifestyle-related factors and insomnia symptoms. Univariate analysis showed that professional title, workplace violence, doctor-patient relationship, chronic pain, chronic disease, physical exercise, perceived stress and anxiety symptoms were statistically significant with insomnia symptoms among doctors (*p* < 0.05). Education, service years, weekly working hours, workplace violence, doctor-patient relationship, chronic pain, chronic disease, physical exercise, perceived stress, anxiety symptoms, and family function were statistically significant with insomnia symptoms among nurses (*p* < 0.05) (Table 2).

**Table 2:** Univariate analysis of insomnia symptoms among doctors and nurses.

Variables	Doctors			Nurses		
	Insomnia symptoms *n*(%)		*P*	Insomnia symptoms *n*(%)		*P*
	No	Yes		No	Yes	
Demographic characteristics						
Gender			0.787			0.221
Male	127(52.9)	113(47.1)		20(58.8)	14(41.2)	
Female	136(51.7)	127(48.3)		224(48.0)	243(52.0)	
Age (years)			0.596			0.222
<30	63(54.8)	52(45.2)		90(53.6)	78(46.4)	
30–40	123(53.2)	108(46.8)		129(47.3)	144(52.7)	
>40	77(49.0)	80(51.0)		25(47.1)	35(58.3)	
Marital status			0.127*			0.073*
Unmarried	85(53.1)	75(46.9)		96(53.3)	84(46.7)	
Married	178(52.5)	161(47.5)		142(45.4)	171(54.6)	
Divorced or loss of spouse	0(0.0)	100(100.0)		6(75.0)	2(25.0)	
Education			0.507			**0.026**
Bachelor degree or below	76(54.7)	63(45.3)		227(47.6)	250(52.4)	
Master degree or above	187(51.4)	177(48.6)		17(70.8)	7(29.2)	
Professional title			**0.021**			0.952
No or junior	88(55.7)	70(44.3)		119(49.4)	122(50.6)	
Intermediate	90(58.4)	64(41.6)		118(48.2)	127(51.8)	
Senior	85(44.5)	106(55.5)		7(46.7)	8(53.3)	
Work-related factors						
Service years			0.207			**<0.001**
≤10	170(54.5)	142(45.5)		165(55.7)	131(44.3)	
>10	93(48.7)	98(51.3)		79(38.5)	126(61.5)	
Weekly working hours			0.117			**<0.001**
≤40	119(56.4)	92(43.6)		194(53.9)	166(46.1)	
>40	144(49.3)	148(50.7)		50(35.5)	91(64.5)	
Number of night shifts per month			0.744			0.170
≤3	155(52.9)	138(47.1)		98(52.7)	88(47.3)	
>3	108(51.4)	102(48.6)		146(46.3)	169(53.7)	
Workplace violence			**<0.001**			**0.002**
No	203(59.2)	140(40.8)		199(52.6)	179(47.4)	
Yes	60(37.5)	100(62.5)		45(36.6)	78(63.4)	
Doctor-patient relationship			**<0.001**			**<0.001**
Good	147(61.5)	92(38.5)		133(58.6)	94(41.4)	
Poor	116(43.9)	148(56.1)		111(40.5)	163(59.5)	
Health and lifestyle-related factors						
Chronic pain			**<0.001**			**<0.001**
No	124(78.5)	34(21.5)		112(79.4)	29(20.6)	
Yes	139(40.3)	206(59.7)		132(36.7)	228(63.3)	
Chronic disease			**<0.001**			**0.003**
No	211(59.6)	143(40.4)		187(53.0)	166(47.0)	
Yes	52(34.9)	97(65.1)		57(38.5)	91(61.5)	
Physical exercise			**0.034**			**0.015**
Never	76(47.8)	83(52.2)		94(44.5)	117(55.5)	
Sometimes	112(50.0)	112(50.0)		105(47.7)	115(52.3)	
Often	75(62.5)	45(37.5)		45(64.3)	25(35.7)	
Perceived stress			**<0.001**			**<0.001**
Low	158(61.0)	101(39.0)		134(58.5)	95(41.5)	
High	105(43.0)	139(57.0)		110(40.4)	162(59.6)	
Anxiety symptoms			**<0.001**			**<0.001**
No	223(59.0)	155(41.0)		202(57.4)	150(42.6)	
Yes	40(32.0)	85(68.0)		42(28.2)	107(71.8)	
Family function			0.168			**<0.001**
Good	185(54.4)	155(45.6)		188(55.1)	153(44.9)	
Moderate or severe dysfunction	78(47.9)	85(52.1)		56(35.0)	104(65.0)	

Significant values are displayed in bold. *Fisher’s exact test.

### Multivariate logistics regression analysis of insomnia symptoms among doctors

Multivariate logistics regression analysis revealed that doctors who had experienced workplace violence were almost 1.631 times more likely to report insomnia symptoms than doctors who had not experienced workplace violence (*OR*: 1.631, 95% *CI*: 1.050–2.532). Doctors with poor doctor-patient relationships were almost 1.603 times more likely to report insomnia symptoms than doctors with good doctor-patient relationships (*OR*: 1.603, 95% *CI*: 1.049–2.450). Doctors with chronic pain were almost 4.134 times more likely to report insomnia symptoms than doctors without chronic pain (*OR*: 4.134, 95% *CI*: 2.579–6.625). Doctors with chronic disease were almost 1.825 times more likely to report insomnia symptoms than doctors without chronic disease (*OR*: 1.825, 95% *CI*: 1.164–2.861). Doctors with anxiety symptoms were almost 2.273 times more likely to report insomnia symptoms than doctors without anxiety symptoms (*OR*: 2.273, 95% *CI*: 1.357–3.807). Thus, workplace violence, doctor-patient relationship, chronic pain, chronic disease, and anxiety symptoms were associated factors with insomnia symptoms in doctors (Table 3).

**Table 3:** Multivariate logistics regression analysis of factors associated with insomnia symptoms among doctors.

Variables	*OR*	95% *CI*	*P*
Professional title			
No or junior	1		
Intermediate	0.726	0.436–1.209	0.219
Senior	1.500	0.917–2.455	0.107
Workplace violence			
No	1		
Yes	1.631	1.050–2.532	**0.029**
Doctor-patient relationship			
Good	1		
Poor	1.603	1.049–2.450	**0.029**
Chronic pain			
No	1		
Yes	4.134	2.579–6.625	**<0.001**
Chronic disease			
No	1		
Yes	1.825	1.164–2.861	**0.009**
Physical exercise			
Never	1		
Sometimes	1.192	0.748–1.900	0.459
Often	0.857	0.490–1.497	0.587
Perceived stress			
Low	1		
High	1.123	0.712–1.771	0.619
Anxiety symptoms			
No	1		
Yes	2.273	1.357–3.807	**0.002**

Significant values are displayed in bold.

### Multivariate logistics regression analysis of insomnia symptoms among nurses

Multivariate logistics regression analysis revealed that nurses with master degree or above were more likely to avoid developing insomnia symptoms (*OR*: 0.301, 95% *CI*: 0.106–0.851). Nurses with more than 10 years of service were almost 1.978 times more likely to have insomnia symptoms than nurses with less than or equal to 10 years of service (*OR*: 1.978, 95% *CI*: 1.304–3.002). Nurses who worked more than 40 h per week were almost 1.694 times more likely to have insomnia symptoms than nurses who worked 40 h per week or less (*OR*: 1.694, 95% *CI*: 1.061–2.705). Nurses with chronic pain were more likely to have insomnia symptoms (*OR*: 5.359, 95% *CI*: 3.241–8.860). Nurses with anxiety symptoms were almost 2.472 times more likely to have insomnia symptoms than nurses without anxiety symptoms (*OR*: 2.472, 95% *CI*: 1.478–4.136). Therefore, education, service years, weekly working hours, chronic pain, and anxiety symptoms were associated factors with insomnia symptoms in nurses (Table 4).

**Table 4:** Multivariate logistics regression analysis of factors associated with insomnia symptoms among nurses.

Variables	*OR*	95% *CI*	*P*
Education			
Bachelor degree or below	1		
Master degree or above	0.301	0.106–0.851	**0.024**
Service years			
≤10	1		
>10	1.978	1.304–3.002	**0.001**
Weekly working hours			
≤40	1		
>40	1.694	1.061–2.705	**0.027**
Workplace violence			
No	1		
Yes	1.252	0.764–2.053	0.373
Doctor-patient relationship			
Good	1		
Poor	1.389	0.896–2.152	0.142
Chronic pain			
No	1		
Yes	5.359	3.241–8.860	**<0.001**
Chronic disease			
No	1		
Yes	1.101	0.700–1.731	0.677
Physical exercise			
Never	1		
Sometimes	1.018	0.655–1.584	0.936
Often	0.731	0.379–1.410	0.350
Perceived stress			
Low	1		
High	1.257	0.807–1.957	0.312
Anxiety symptoms			
No	1		
Yes	2.472	1.478–4.136	**0.001**
Family function			
Good	1		
Moderate or severe dysfunction	1.288	0.806–2.058	0.290

Significant values are displayed in bold.

## Discussion

This cross-sectional study included 503 doctors and 501 nurses. The results showed that the prevalence of insomnia symptoms was high among doctors and nurses in third-grade class-A general hospitals under the context of high prevalence of multiple infectious diseases. The prevalence of insomnia symptoms was 47.7% in doctors and 51.3% in nurses. The prevalence of insomnia symptoms was higher among nurses than among doctors, a finding consistent with the results of previous studies (42, 43). Furthermore, the prevalence of insomnia symptoms among doctors in this study was equivalent to that in Turkey (44.1%) (44). The prevalence of insomnia symptoms among nurses in this study was higher than that in Rio de Janeiro, Brazil (34.3%) and lower than that in Turkey (60.6%) (14, 44). The possible reasons for this were differences in study methods, samples, and contexts. Overall, doctors and nurses are experiencing the effects of insomnia symptoms. Therefore, it is necessary to screen doctors and nurses regularly for insomnia symptoms and provide them with ongoing support. Notably, this study also identified education, service years, weekly working hours, chronic pain, and anxiety symptoms were associated factors with insomnia symptoms in nurses. Workplace violence, doctor-patient relationship, chronic pain, chronic disease, and anxiety symptoms were associated factors with insomnia symptoms in doctors.

Years of education was closely related to sleep disturbances (43). In this study, more insomnia symptoms were observed among nurses with a bachelor degree or below of education. This is similar to previous findings among medical staff that were education level associated with insomnia symptoms (45). This is also consistent with previous studies in the general Chinese population, which showed that low education level was associated with high insomnia symptoms (46). The possible reason for this difference is that nurses with low education level felt inadequate in their nursing ability. Therefore, nurses with low education need more intervention support for insomnia symptoms.

Studies showed that women health workers who have worked for more than 10 years were vulnerable to stress, depression and anxiety (47). Long working years was a risk factor for insomnia in medical staff (48). The present study, similar to previous reports, found that nurses with more than 10 years of service were more likely to develop insomnia symptoms. One possibility is that nurses with longer service years may face more occupational fatigue. Therefore, more attention should be paid to nurses with longer service years in the development of insomnia interventions. Evidence suggested that work overload could predict levels of insomnia (49). This study found that nurses working more than 40 h per week were more likely to experience insomnia symptoms. This may be because nurses are often required to work shifts and longer working hours result in insufficient recovery time between shifts. Insufficient recovery time has been shown to be associated with worse sleep (50). Therefore, reasonable arrangement of nurses’ working time and improvement of nurses’ working efficiency should be paid attention to.

Systematic reviews and meta-analyses have shown a direct relationship between exposure to violence and sleep problems among workers (51). Longitudinal surveys in Swedish suggested an association between the occurrence of workplace violence and the subsequent appearance of sleep disturbances (52). Prevention of workplace violence may improve sleep quality (53). This study also demonstrated that workplace violence was associated with insomnia symptoms in doctors. In China, most doctors have experienced various kinds of violence from patients or their families (15). The current study found that the prevalence of workplace violence in doctors was 31.8%. Workplace violence against doctors has become a public health problem (54). Further research on doctor workplace violence is needed so that effective measures can be taken to prevent doctor workplace violence. This may not only reduce various harms caused by workplace violence, but also reduce the incidence of insomnia symptoms. In China, the doctor-patient relationship is fragile, and most patients do not trust doctors (54). An important reason is that most doctors focus on dealing with technical problems and ignore the humanistic aspects of care (15). In addition, the public is less satisfied with the current doctor-patient relationship (55). This study found that the doctor-patient relationship was an important influencing factor of insomnia symptoms in doctors. Therefore, it is very important to improve the doctor-patient relationship. This requires the engagement and increased effort of healthcare workers, patients and managers.

This study shed new light on a variable rarely mentioned in the previous literature, that was, chronic pain was associated factor for insomnia symptoms among doctors and nurses. This is similar to previous reports in other populations in which frequent pain was significantly associated with an increased prevalence of insomnia symptoms (56). Notably, this study found that 68.6 and 71.9% of doctors and nurses, respectively, had pain in at least one body part for more than 1 month. These findings highlight the importance of assessing and managing pain among doctors and nurses. Insomnia was highly prevalent in patients with chronic diseases (57). This study found that doctors with chronic disease were more likely to report insomnia symptoms. This result is similar to previous findings in which the occurrence of chronic diseases had a statistically significant effect on the severity of negative mental health symptoms such as insomnia (58). The underlying symptoms of chronic diseases themselves and treatment-related factors may interfere with sleep and contribute to the high prevalence of insomnia in this population (59). Therefore, more attention should be paid to doctors with chronic diseases and effective measures should be developed.

There was a bidirectional relationship between anxiety and sleep disturbances (43). The current study found that anxiety symptoms were significantly associated with insomnia symptoms among doctors and nurses. This is supported by previous findings in which anxiety level was an influencing factor for insomnia among front-line medical staff (60). Thus, early recognition of signs of insomnia symptoms is crucial.

Although this study presents some important findings, there are some limitations that need to be mentioned. First, this study was a cross-sectional survey and could not find causal relationships between variables. Therefore, longitudinal research is a direction worthy of attention in the future. Second, we conducted the survey in only one city, which is very small compared to the Chinese population of doctors and nurses. The results can only be generalized to regions with the same background. Therefore, future studies with more regions and larger samples are needed. Finally, this study only focused on third-grade class-A general hospitals and ignored specialized hospitals and primary hospitals. Future studies are recommended to be carried out in hospitals of different nature.

## Conclusion

In conclusion, this study demonstrated a high prevalence of insomnia symptoms among doctors and nurses in third-grade class-A general hospitals. Close monitoring and early intervention are essential. This study also identified workplace violence, doctor-patient relationship, chronic pain, chronic disease, and anxiety symptoms were associated factors with insomnia symptoms in doctors. Education, service years, weekly working hours, chronic pain, and anxiety symptoms were associated factors with insomnia symptoms in nurses. This can help doctors and nurses to develop precise interventions for insomnia symptoms, resulting in benefits for doctor and nurse long-term health outcomes, quality of care, and the overall economy.

## Data Availability

The raw data supporting the conclusions of this article will be made available by the authors, without undue reservation.
